# Femoral Artery Closure Devices vs Manual Compression During Cardiac Catheterization and Percutaneous Coronary Intervention

**DOI:** 10.1016/j.jscai.2022.100370

**Published:** 2022-06-29

**Authors:** Rolf P. Kreutz, Sujoy Phookan, Hamid Bahrami, Anjan K. Sinha, Jeffrey A. Breall, George E. Revtyak, Georges Ephrem, Joseph R. Zenisek, Kyle A. Frick, Ziad A. Jaradat, Ibrahim S. Abu Romeh, Brian A. O’Leary, Hamza Z. Ansari, Andrew D. Ferguson, Kevin E. Zawacki, Mohammad Z. Hoque, Ali F. Iqtidar, Nathan D. Lambert, Elisabeth von der Lohe

**Affiliations:** a Division of Cardiovascular Medicine, Indiana University School of Medicine/Indiana University Health Methodist, Indianapolis, Indiana; b Division of Cardiology, Indiana University Health West, Avon, Indiana; c Division of Cardiology, Indiana University Health Ball Memorial, Muncie, Indiana; d Division of Cardiology, Indiana University Health Bloomington, Bloomington, Indiana; e Division of Cardiology, Indiana University Health Arnett, Lafayette, Indiana; f Division of Cardiology, Indiana University Health Saxony/North, Fishers/Carmel, Indiana

**Keywords:** bleeding, cardiac catheterization, percutaneous coronary intervention

## Abstract

**Background:**

Femoral arterial access remains widely used despite recent increase in radial access for cardiac catheterization and percutaneous coronary intervention (PCI). Various femoral artery closure devices have been developed and are commonly used to shorten vascular closure times, with variable rates of vascular complications observed in clinical trials. We sought to examine the rates of contemporary outcomes during diagnostic catheterization and PCI with the most common femoral artery closure devices.

**Methods:**

We identified patients who had undergone either diagnostic catheterization alone (*n* = 14,401) or PCI (*n* = 11,712) through femoral artery access in the Indiana University Health Multicenter Cardiac Cath registry. We compared outcomes according to closure type: manual compression, Angio-Seal, Perclose, or Mynx. Access complications and bleeding outcomes were measured according to National Cardiovascular Data ​Registry standard definitions.

**Results:**

The use of any vascular closure device as compared to manual femoral arterial access hold was associated with a significant reduction in vascular access complications and bleeding events in patients who underwent PCI. No significant difference in access-site complications was observed for diagnostic catheterization alone. Among closure devices, Perclose and Angio-Seal had a lower rate of hematoma than Mynx.

**Conclusions:**

The use of femoral artery access closure devices is associated with a reduction in vascular access complication rates as compared to manual femoral artery compression in patients who undergo PCI.

## Highlights

• Retrospective comparison was performed of femoral artery closure devices.
• Lower risk of bleeding was observed with use of vascular closure device in PCI.
• No difference in access site bleeding was seen in diagnostic catheterization.

## Central Illustration

**Figure:**
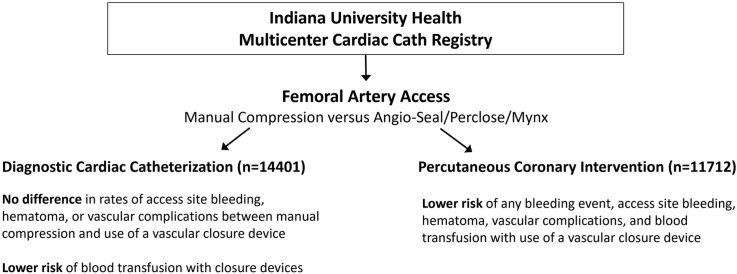

## Introduction

Despite the increase in radial artery access for cardiac catheterization and percutaneous coronary intervention (PCI), femoral artery puncture and canulation remains a standard arterial access form. While rare, complications after removal of arterial access sheaths can occur and include continued access site bleeding, formation of a hematoma, arterial pseudoaneurysm, retroperitoneal hemorrhage, or acute thrombotic vessel occlusion. Various arterial closure devices have been developed over time, some of which are no longer in use.^1^ Device approval trials usually focused on early ambulation and device safety and usually contain relatively small numbers of patients.^2^, ^3^, ^4^ Postapproval studies have been conducted comparing specific closure devices individually or against manual compression.^5^^,^^6^ Meta-analyses have suggested similar complication rates to manual compression alone with vascular closure devices; however, there is significant heterogeneity in studies over time.^7^, ^8^, ^9^ We hypothesized that in the current era, femoral artery closure devices are associated with improved outcomes as compared to manual compression when used during PCI.

## Methods

### Study objective

The objective of the current study was to examine rates of vascular access site complications and clinical outcomes in contemporary clinical practice among patients who underwent diagnostic cardiac catheterization or PCI using femoral artery access and to compare vascular access closure devices with standard manual compression.

### Patient population

The study examined data from the Indiana University Health Multicenter Cath Registry study, which included patients who underwent cardiac catheterization and PCI at 7 participating hospitals in Indiana, USA, between 2015 and 2021 (IU Health Methodist, West, North, Saxony, Ball, Bloomington, and Arnett). Indiana University Institutional Review Board approval was obtained for the study.

### Study design and endpoint definition

We identified patients who underwent either diagnostic catheterization or PCI through the femoral artery with closure of access site by manual compression, using an Angio-Seal device (Angio-Seal VIP; Terumo), using a Perclose device (Perclose A-T or Perclose ProGlide; Abbott), or using a Mynx device (Mynx ACE or MYNXGRIP; Cordis). Demographics, comorbidities, and clinical variables, as well as procedural details, were prospectively captured as defined by the National Cardiovascular Data ​Registry (NCDR) Cath PCI database.^10^ Information on access sheath size was not available in the registry at the patient level. Information on distribution of access sheath sizes used for cardiac catheterization was available through query of inventory data between 2020 and 2021. The use of fluoroscopy for femoral artery access is standard in our institutions, and femoral angiography is always performed prior to deployment of vascular closure devices. The use of ultrasound or micropuncture needles was variable at the discretion of the operator, and information on their use was not collected prospectively.

Study endpoint definitions were used as defined by NCDR CathPCI during the index hospitalization. Endpoints included access site bleeding (defined as any access site bleeding with hemoglobin [Hgb] drop ≥3 ​g/dL, blood transfusion, or requiring surgery/intervention), hematoma at access site (defined as hematoma with Hgb drop ≥3 ​g/dL, blood transfusion, or requiring surgery/intervention), any blood transfusion during hospital stay, retroperitoneal hemorrhage (defined as any documented retroperitoneal bleeding with Hgb drop ≥3 ​g/dL, blood transfusion, or requiring surgery/intervention), other vascular complications requiring intervention, and any bleeding event within 72 ​hours (defined as any bleeding event with Hgb drop ≥3 ​g/dL, blood transfusion, or requiring surgery/intervention).^10^ Clinical events were adjudicated retrospectively through review of medical records and source data. Analysis was conducted separately for diagnostic catheterizations (without ad hoc PCI) and PCI cases (including planned PCI and diagnostic catheterizations with ad hoc PCI).

### Statistical analysis

Baseline variables were compared between groups using Pearson χ^2^ test. Continuous data were compared using analysis of variance for multiple groups. Fisher’s exact test was used to compare event rates. Testing was performed 2-sided with *P* ​< ​.05 considered significant. Statistical analysis was performed with the use of SPSS software, version 28.0 (IBM Corp).

## Results

We identified 26,113 subjects who underwent either diagnostic cardiac catheterization (*n* = 14,401) or PCI (*n* = 11,712) using femoral arterial access in the Indiana University Health Multicenter Cardiac Cath Registry. During the study period, radial artery access was used in 46% of diagnostic catheterizations and in 42% of PCI cases. The majority of patients had femoral arterial closure using manual compression alone (*n* ​= 13,462). The remaining patients had closure with either Angio-Seal (*n* = 5786), Perclose (*n* = 2143), or Mynx (*n* = 4721). The proportion of subjects who had a closure device placed was higher among patients who underwent PCI (63%) than those who had a diagnostic catheterization only (36%). Angio-Seal and Perclose were more often used in PCI than Mynx which was used equally during diagnostic and PCI cases. Patients who received a closure device were more likely male and were less likely to be in cardiogenic shock or on mechanical support. As expected, clinical variables were not evenly balanced between manual femoral access closure and the various closure devices (Tables 1 and 2).

**Table 1:** Baseline demographics, clinical, and procedural characteristics of the patients who underwent diagnostic catheterization alone.

Diagnostic catheterizations (*n* ​= ​14,401)	Femoral manual hold (*n* ​= ​9183)	Angio-Seal (*n* ​= ​2151)	Perclose (*n* ​= ​665)	Mynx (*n* ​= ​2402)	Any closure device (*n* ​= ​5218)	*P* value, all	*P* value (comparison between closure devices)	*P* value (comparison any closure device vs manual)
Age, y	63.6 ​± ​13	62.3 ​± ​11.4	65.3 ​± ​12.2	65.2 ​± ​12.5	64 ​± ​12.1	<.001	<.001	.051
Women	4073 (44.4%)	810 (37.7%)	257 (38.6%)	1119 (46.6%)	2186 (41.9%)	<.001	<.001	<.01
Men	5109 (55.6%)	1341 (62.3%)	408 (61.4%)	1283 (53.4%)	3032 (58.1%)			
White	7911 (86.1%)	1934 (89.0%)	622 (93.5%)	2211 (92%)	4767 (91%)	<.001	.004	<.001
Black	1106 (12%)	186 (8.6%)	33 (5%)	161 (6.7%)	380 (7.3%)	<.001	.002	<.001
Asian	93 (1%)	18 (0.8%)	8 (1.2%)	15 (0.6%)	41 (0.8%)	.28	.3	.17
Hispanic	118 (1.3%)	37 (1.7%)	4 (0.6%)	29 (1.2%)	70 (1.3%)	.19	.07	.76
Body mass index, kg/m^2^	30.1 ​± ​15.9	30.7 ​± ​6.6	30.8 ​± ​7.1	31.5 ​± ​7.2	31.1 ​± ​7	<.001	<.001	<.001
Diabetes mellitus	3452 (37.6%)	925 (43%)	265 (39.8%)	976 (40.6%)	2166 (41.5%)	<.001	.17	<.001
Hypertension	7227 (78.7%)	1500 (69.7%)	537 (80.8%)	1964 (81.8%)	4001 (76.7%)	<.001	<.001	<.01
Hyperlipidemia	6975 (76%)	1261 (58.6%)	513 (77.1%)	1912 (79.6%)	3686 (70.6%)	<.001	<.001	<.001
Peripheral vascular disease	890 (9.7%)	142 (6.6%)	73 (11%)	255 (10.6%)	470 (9%)	<.001	<.001	.18
Prior stroke	787 (8.6%)	144 (6.7%)	53 (8%)	203 (8.5%)	400 (7.7%)	.04	.08	.06
Prior PCI	2470 (26.9%)	591 (27.5%)	206 (31%)	883 (36.8%)	1680 (32.2%)	<.001	<.001	<.001
Prior CABG	1335 (14.5%)	299 (13.9%)	138 (20.8%)	514 (21.4%)	951 (18.2%)	<.001	<.001	<.001
Chronic lung disease	1487 (24.6%)	177 (12.6%)	93 (18.6%)	306 (20.6%)	576 (11%)	<.001	<.001	<.001
End-stage renal disease	541 (5.9%)	213 (9.9%)	27 (4.1%)	124 (5.2%)	364 (7%)	<.001	<.001	.01
Mechanical ventricular support	146 (1.6%)	8 (0.4%)	1 (0.2%)	6 (0.2%)	15 (0.3%)	<.001	.24	<.001
Cardiogenic shock	189 (2.1%)	17 (0.8%)	10 (1.5%)	16 (0.7%)	43 (0.8%)	.002	.1	<.001
Cardiac arrest	196 (2.1%)	22 (1%)	16 (2.4%)	31 (1.3%)	69 (1.3%)	<.001	.02	<.001
STEMI	152 (1.7%)	40 (1.9%)	38 (5.7%)	21 (0.9%)	99 (1.9%)	<.001	<.001	.29
NSTEMI	899 (9.8%)	132 (6.1%)	80 (12%)	215 (9%)	427 (8.2%)	<.001	<.001	.001

CABG, coronary artery bypass grafting; NSTEMI, non-ST elevation myocardial infarction; PCI, percutaneous coronary intervention; STEMI, ST-elevation myocardial infarction.

**Table 2:** Baseline demographics, clinical, and procedural characteristics of the patients who underwent percutaneous coronary intervention.

Percutaneous coronary interventions (*n* ​= ​11,712)	Femoral manual hold (*n* ​= ​4280)	Angio-Seal (*n* ​= ​3635)	Perclose (*n* ​= ​1478)	Mynx (*n* ​= ​2319)	Any closure device (*n* ​= ​7432)	*P* value, all	*P* value (comparison between closure devices)	*P* value (comparison any closure device vs manual)
Age, y	66.1 ​± ​12.4	63.7 ​± ​11.8	64.2 ​± ​12.6	66.7 ​± ​11.5	64.7 ​± ​12	<.001	<.001	<.001
Women	1531 (35.8%)	1185 (32.6%)	426 (28.8%)	860 (37.1%)	2471 (33.2%)	<.001	<.001	<.01
Men	2749 (64.2%)	2450 (67.4%)	1052 (71.2%)	1459 (62.9%)	4961 (66.8%)			
White	3976 (92.9%)	3164 (87%)	1384 (93.6%)	2114 (91.2%)	6662 (89.6%)	<.001	<.001	<.001
Black	249 (5.8%)	397 (10.9%)	55 (3.7%)	153 (6.6%)	605 (8.1%)	<.001	<.001	<.001
Asian	29 (0.7%)	45 (1.2%)	29 (2%)	31 (1.3%)	105 (1.4%)	.28	.13	<.001
Hispanic	44 (1%)	52 (1.5%)	16 (1.1%)	36 (1.6%)	104 (1.4%)	.27	.47	.08
Body mass index, kg/m^2^	30 ​± ​9	31.1 ​± ​7.2	30.4 ​± ​7.3	30.7 ​± ​7.3	30.8 ​± ​7.3	<.001	.004	<.001
Diabetes mellitus	1858 (43.4%)	1648 (45.3%)	574 (38.9%)	1108 (47.8%)	3330 (44.8%)	<.001	<.001	.14
Hypertension	3515 (82.1%)	2952 (81.2%)	1146 (77.5%)	2089 (90.1%)	6187 (83.2%)	<.001	<.001	.12
Hyperlipidemia	3281 (76.7%)	2721 (74.9%)	1114 (75.4%)	2069 (89.2%)	5904 (79.4%)	<.001	<.001	<.001
Prior stroke	683 (16%)	397 (10.9%)	150 (10.1%)	331 (14.3%)	878 (11.8%)	<.001	<.001	<.001
Peripheral vascular disease	795 (18.6%)	438 (12%)	166 (11.2%)	454 (19.6%)	1058 (14.2%)	<.001	<.001	<.001
Prior PCI	1847 (43.2%)	1590 (43.7%)	573 (38.8%)	1362 (58.8%)	3525 (47.4%)	<.001	<.001	<.001
Prior CABG	891 (20.8%)	657 (18.1%)	257 (17.4%)	642 (27.7%)	1556 (20.9%)	<.001	<.001	.88
Chronic lung disease	821 (19.2%)	541 (14.9%)	201 (13.6%)	351 (15.1%)	1093 (14.7%)	<.001	.39	<.001
End-stage renal disease	223 (5.2%)	316 (8.7%)	49 (3.3%)	68 (2.9%)	433 (5.8%)	<.001	<.001	.16
Mechanical ventricular support	383 (8.9%)	83 (2.3%)	55 (3.7%)	24 (1%)	162 (2.2%)	<.001	<.001	<.001
Cardiogenic shock	303 (7.1%)	48 (1.3%)	21 (1.4%)	20 (0.9%)	89 (1.2%)	.002	.19	<.001
Cardiac arrest	291 (6.8%)	90 (2.5%)	38 (2.6%)	27 (1.2%)	155 (2.1%)	<.001	<.001	<.001
STEMI	1368 (32%)	763 (21%)	445 (30.1%)	189 (8.2%)	1397 (18.8%)	<.001	<.001	<.001
NSTEMI	1328 (31%)	1117 (30.7%)	418 (28.3%)	580 (25%)	2115 (28.5%)	<.001	<.001	<.01
Heparin	3219 (75.3%)	3106 (85.5%)	984 (66.6%)	1364 (58.8%)	5454 (73.4%)	<.001	<.001	.029
Bivalirudin	1254 (29.3%)	612 (16.8%)	569 (38.5%)	987 (42.6%)	2168 (29.2%)	<.001	<.001	.89
Enoxaparin	300 (7%)	250 (6.9%)	136 (9.2%)	225 (9.7%)	611 (8.2%)	<.001	<.001	.018
GP IIb/IIIa inhibitor	1146 (26.8%)	403 (11.1%)	164 (11.1%)	190 (8.2%)	757 (10.2%)	<.001	<.001	<.001
Cangrelor	66 (4%)	26 (1.4%)	5 (0.8%)	4 (0.7%)	35 (0.5%)	<.001	.29	<.001
Clopidogrel	1958 (45.8%)	1784 (49.1%)	796 (53.9%)	1374 (59.3%)	3954 (53.2%)	<.001	<.001	<.001
Prasugrel	430 (10.1%)	328 (9%)	134 (9.1%)	241 (10.4%)	703 (9.5%)	.22	.18	.3
Ticagrelor	1556 (36.4%)	1451 (39.9%)	495 (33.5%)	516 (22.3%)	2462 (33.1%)	<.001	<.001	<.001

CABG, coronary artery bypass grafting; GP, glycoprotein; NSTEMI, non-ST elevation myocardial infarction; PCI, percutaneous coronary intervention; STEMI, ST-elevation myocardial infarction.

**Figure 1:**
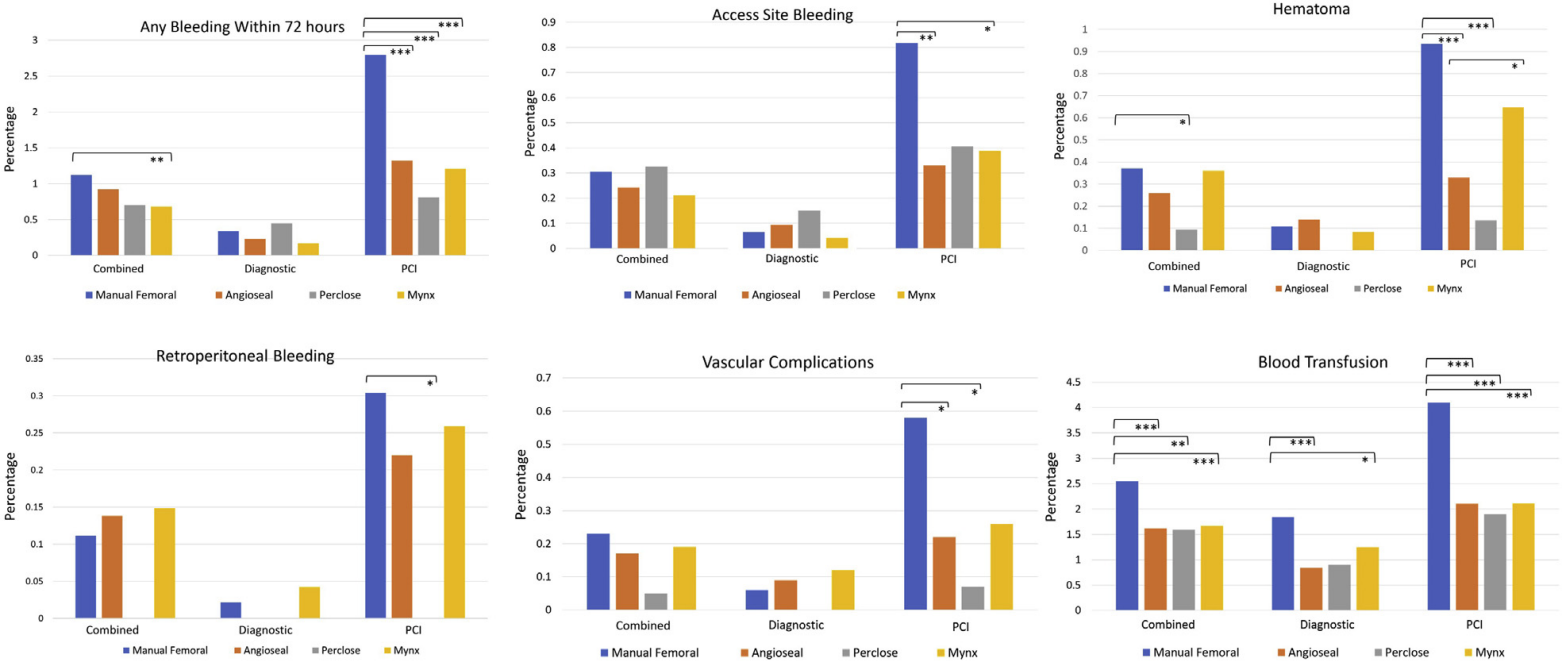
Femoral arterial access site complications and bleeding events according to manual femoral artery compression alone vs Angio-Seal, Perclose, or Mynx closure devices. ∗*P* ​< ​.05, ∗∗*P* < .01, ∗∗∗*P* < .001. PCI, percutaneous coronary intervention.

During diagnostic cardiac catheterization, the risk of any bleeding event within 72 ​hours, bleeding at access site, retroperitoneal hemorrhage, and hematoma was not significantly different between manual femoral closure and the use of a closure device (Table 3). However, the odds of requiring any blood transfusion after a diagnostic catheterization was lower with the use of Angio-Seal, Mynx, or any vascular closure device than with manual hold (odds ratio [OR], 0.56; 95% confidence interval [CI], 0.41-0.76) (Table 3 and Figure 1).

**Table 3:** Bleeding events among patients who underwent diagnostic catheterization alone.

Diagnostic catheterization bleeding events	Femoral manual hold	Angio-Seal	Perclose	Mynx	Any closure device	Odds ratio (95% CI) (Angio-Seal vs manual)	*P* value	Odds ratio (95% CI) (Perclose vs manual)	*P* value	Odds ratio (95% CI) (Mynx vs manual)	*P* value	Odds ratio (95% CI) (any closure device vs manual)	*P* value
Any bleeding within 72 ​h	31/9183 (0.34%)	5/2151 (0.23%)	3/665 (0.45%)	4/2402 (0.17%)	12/5218 (0.22%)	0.69 (0.27-1.77)	.53	1.34 (0.41-4.4)	.5	0.49 (0.17-1.4)	.21	0.68 (0.35-1.33)	.27
Bleeding at access site	6/9183 (0.07%)	2/2151 (0.09%)	1/665 (0.2%)	1/2402 (0.04%)	4/5218 (0.08%)	1.42 (0.29-7)	.65	2.3 (0.28-19)	.38	0.64 (0.08-5.3)	1.0	1.17 (0.33-4.2)	.76
Hematoma	10/9183 (0.1%)	3/2151 (0.14%)	0/665 (0%)	2/2402 (0.09%)	5/5218 (0.1%)	1.28 (0.35-4.7)	.72	NA	1.0	0.76 (0.17-3.5)	1.0	0.88 (0.3-2.6)	1.0
Retroperitoneal bleeding	2/9183 (0.02%)	0/2151 (0%)	0/665 (0%)	1/2402 (0.04%)	1/5218 (0.02%)	NA	1.0	NA	1.0	1.72 (0.16-19)	.53	0.88 (0.08-9.7)	1.0
Vascular complication requiring intervention	6/9183 (0.06%)	2/2151 (0.09%)	0/665 (0%)	3/2402 (0.12%)	5/5218 (0.1%)	1.42 (0.29-7.1)	.65	NA	1.0	1.91 (0.48-7.7)	.4	1.47 (0.45-4.8)	.54
Blood transfusion	169/9183 (1.84%)	18/2151 (0.84%)	6/665 (0.9%)	30/2402 (1.25%)	54/5218 (1%)	0.45 (0.28-0.73)	.001	0.49 (0.21-1.1)	.09	0.67 (0.46-0.99)	.05	0.56 (0.41-0.76)	<.001

CI, confidence interval; NA, not available.

During PCI, the risk of any bleeding event within 72 ​hours was significantly lower with all vascular closure devices (1.2%) than that with manual hold (2.8%) (OR, 0.42; 95% CI, 0.32-0.55) (Table 4 and Figure 1). Bleeding at access site was significantly lower with use of Angio-Seal and Mynx, but not with Perclose (Table 3 and Figure 1). Hematoma at access site occurred less frequently with the use of Angio-Seal (0.3%) and Perclose (0.1%), but not with Mynx (0.65%) as compared to manual compression (0.9%) (Table 3 and Figure 1). No cases of retroperitoneal hemorrhage were observed with the use of a Perclose device, which was a significant reduction in incidence as compared to manual compression (Figure 1). Incidence of retroperitoneal hemorrhage was not significantly lower with Angio-Seal (0.2%) and Mynx (0.25%) than that with manual compression (0.3%) during PCI. Mynx was associated with a higher risk of hematoma (*P* < .001) in PCI cases when compared to Angio-Seal and Perclose, and the risk of retroperitoneal hemorrhage was highest for Mynx among the closure devices (Figure 1). The risk of other vascular complications requiring intervention was lower with Angio-Seal and Perclose than that with manual compression in PCI cases, but not with Mynx (Table 3 and Figure 1). Overall blood transfusion was less common with the use of any vascular closure device during PCI than that with manual compression (OR, 0.5; 95% CI, 0.4-0.62) (Table 4 and Figure 1). Institutional inventory use data showed that diagnostic catheterizations using femoral artery access were performed most commonly with 4F (50.3%), followed by 5F (30.9%), and 6F (18.8%) sheath sizes. Femoral artery access sheath sizes for PCI were most commonly 6F (80.6%), followed by 7F (7.8%), 8F (4.4%), and 5F (4.1%).

**Table 4:** Bleeding events among patients who underwent percutaneous coronary intervention.

Percutaneous coronary interventions bleeding events	Femoral manual hold	Angio-Seal	Perclose	Mynx	Any closure device	Odds ratio (95% CI) (Angio-Seal vs manual)	*P* value	Odds ratio (95% CI) (Perclose vs manual)	*P* value	Odds ratio (95% CI) (Mynx vs manual)	*P* value	Odds ratio (95% CI) (any closure device vs manual)	*P* value
Any bleeding within 72 ​h	120/4280 (2.8%)	48/3635 (1.32%)	12/1478 (0.81%)	28/2319 (1.21%)	88/7432 (1.2%)	0.46 (0.33-0.65)	<.001	0.28 (0.16-0.52)	<.001	0.42 (0.28-0.64)	<.001	0.42 (0.32-0.55)	<.001
Bleeding at access site	35/4280 (0.82%)	12/3635 (0.33%)	6/1478 (0.4%)	9/2319 (0.39%)	27/7432 (0.4%)	0.4 (0.21-0.78)	<.01	0.49 (0.21-1.2)	.15	0.47 (0.28-0.99)	.04	0.44 (0.27-0.73)	.001
Hematoma	40/4280 (0.93%)	12/3635 (0.3%)	2/1478 (0.14%)	15/2319 (0.65%)	29/7432 (0.39%)	0.35 (0.18-0.67)	.001	0.14 (0.04-0.6)	<.001	0.69 (0.38-1.25)	.26	0.42 (0.26-0.67)	<.001
Retroperitoneal bleeding	13/4280 (0.3%)	8/3635 (0.22%)	0/1478 (0%)	6/2319 (0.3%)	14/7432 (0.19%)	0.72 (0.3-1.7)	.52	NA	.03	0.85 (0.32-2.2)	.82	0.62 (0.29-1.3)	.23
Vascular complication requiring intervention	25/4280 (0.58%)	8/3635 (0.22%)	1/1478 (0.07%)	6/2319 (0.26%)	15/7432 (0.2%)	0.38 (0.17-0.83)	.014	0.12 (0.02-0.85)	.01	0.44 (0.18-1.1)	.09	0.34 (0.18-0.65)	<.01
Blood transfusion	174/4280 (4.1%)	76/3635 (2.1%)	28/1478 (1.9%)	49/2319 (2.11%)	153/7432 (2.1%)	0.5 (0.38-0.66)	<.001	0.46 (0.3-0.68)	<.001	0.51 (0.37-0.7)	<.001	0.5 (0.4-0.62)	<.001

CI, confidence interval; NA, not available.

## Discussion

Manual vessel compression has been the original standard for femoral artery access removal since the inception of invasive cardiology; however, several generations of intravascular and extravascular closure devices have been developed to facilitate arterial access closure.^1^ While complications continue to occur, rates of vascular access bleeding have substantially decreased both for manual vessel compression as well as vascular closure device use over the last 3 ​decades. This is in part due to the use of smaller sheath sizes down to 4F for diagnostic catheterizations with the use of injection-assist devices, as well as less frequent use of glycoprotein IIb/IIIa inhibitors in treatment of acute coronary syndrome and during PCI. Many of the prospective studies conducted with Perclose and Angio-Seal in PCI several decades ago included routinely larger femoral artery sheath sizes of ≥8F.^9^

Also, increased attention to bleeding complications as drivers of adverse clinical outcomes has sharpened clinical education with a stronger focus given to optimal femoral artery access.^11^^,^^12^ This now includes standard use of fluoroscopy, micropuncture needle kits, and point-of-care ultrasound to optimally guide access in the common femoral artery and minimize off-target vessel access.^12^^,^^13^ The vascular closure devices most commonly used for standard femoral artery access closure with sheath sizes between 5F and 8F are the Angio-Seal, the Perclose ProGlide, and the MYNXGRIP. The Angio-Seal device uses an intravascular absorbable polymer anchor with an extravascular attached collagen sponge connected by an absorbable self-tightening suture in either 6F or 8F sheath size.^3^ The Perclose ProGlide is an endovascular suture-mediated percutaneous closure device approved for 5-8F sheath sizes but can be used in up to 21F arterial sheath sizes if deployed before upsizing of catheter sheaths beyond 8F and if 2 devices are used (preclose technique).^4^ The MYNXGRIP is an absorbable extravascular water-soluble synthetic sealant made of polyethylene glycol delivered with the use of a removable intravascular balloon and standard 5-7F access sheaths.^2^ All 3 vascular closure devices have restrictions on their labels related to use in off-target vessel zones, and all have restrictions for small vessel caliber (Angio-Seal VIP < 4 ​mm, Perclose ProGlide < 5 ​mm, MYNXGRIP < 5 ​mm).^2^, ^3^, ^4^ Arterial closure devices are usually not applied in favor of manual compression in cases where there is significant atherosclerotic disease present at the puncture site, if the puncture is near or below the bifurcation of the superficial femoral artery and profunda femoris artery, or if the vessel size is too small. Earlier trials enrolling small numbers of patients with the devices focused on early ambulation as endpoints and included variable rates of vascular complications, but not significantly different from manual compression.^6^^,^^8^^,^^9^^,^^14^ In clinical practice, vascular closure devices continue to be used frequently to shorten bedrest time and reduce the risk of access bleeding, yet limited data are available comparing the efficacy and safety of the various devices.^7^^,^^8^ In prior studies, vascular complications with femoral artery access were more likely to occur in older patients and those with larger access sheaths (≥7F).^15^

The data from our study indicate that the use of a vascular closure device does not significantly reduce access site bleeding and risk of hematoma when compared to manual artery compression alone for patients who undergo diagnostic catheterization alone (Central Illustration). This is not very surprising given that most diagnostic cardiac catheterizations in our institutions using femoral artery access are conducted using 4F or 5F access sheaths, which carry a lower risk of bleeding in the absence of anticoagulation. In contrast, patients who underwent PCI and thus usually require larger sheath sizes and periprocedural anticoagulation with heparin or bivalirudin demonstrated a significant reduction in access site bleeding, risk of hematoma, as well as lower risk of vascular complications with the use of a closure device (Central Illustration). In addition, the odds of requiring a blood transfusion were lower with the use of a closure device although the rate of blood transfusion was higher than the incidence of access site bleeding events overall, suggesting that many transfusion events are caused by concomitant medical conditions (chronic anemia, gastrointestinal blood loss, etc.) or bleeding below the required threshold of Hgb drop to qualify as an access site bleed. Among closure devices, significant differences are seen for some complications. Mynx was found to have higher risk of hematoma among patients who underwent PCI than Angio-Seal and Perclose. This may be due to the design of the Mynx closure device, which, beyond the passive attachment of the polyethylene glycol to the vessel wall and sealing with fibrin, does not have any remaining fixed attachment to the arteriotomy location. The Mynx plug can thus more easily be displaced by pulling of subcutaneous or muscle structures that may also be joined to the sealant when the patient mobilizes, which is not possible with a suture-based closure device such as Perclose and less likely with an endovascular anchor-based device such as the Angio-Seal. An earlier version of the Mynx device used between 2011 and 2013 was also associated with increased risk of vascular complications in the NCDR/CathPCI registry.^16^ As a noteworthy difference, none of the Perclose closure patients in our registry suffered a retroperitoneal hematoma, which was a significant difference compared to manual compression. Vascular closure devices in our study were more often used in younger patients during PCI and less in patients with other high-risk comorbidities such as cardiogenic shock, prior stroke, peripheral vascular disease, or for a ST-elevation myocardial infarction indication.

**Central Illustration:**
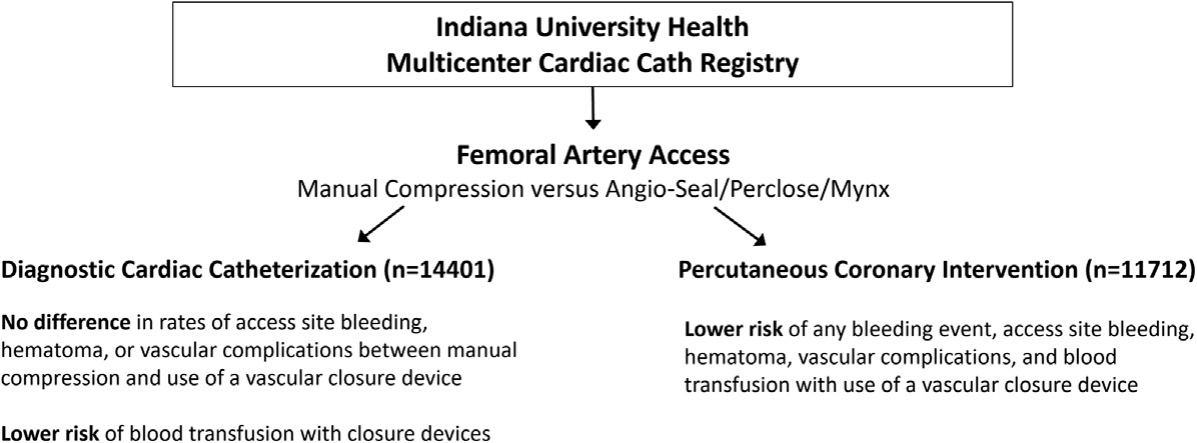
Femoral artery access closure comparison and main outcomes.

Prior studies have shown a consistent association between bleeding events of the same definition as in our study with reduced survival after PCI,^17^ and avoidance of access site bleeding has been the main reason behind the adoption of a “radial-first” approach as recommended in both European and US clinical practice guidelines.^18^^,^^19^ Therefore, successful deployment of a vascular closure device after femoral access PCI is likely of significant clinical benefit and should be recommended. This approach is supported by the reduction in arterial access bleeding events in the “Safety and Efficacy of Femoral Access vs Radial Access in ST-Segment Elevation Myocardial Infarction” (SAFARI-STEMI) study compared to other trials that compared radial vs femoral artery access and in which 68% of femoral access patients had a vascular closure device placed.^20^

Limitations of our study include the nonrandomized retrospective nature of the registry study and the limitation in covariates and outcome data to those collected in the registry. It is possible that the manual compression group was in part not eligible for a closure device due to more severe atherosclerotic disease or that other confounders not included in the analysis could have contributed to differences in outcomes. In addition, we did not have patient-level information on sheath size to include in the analysis.

In conclusion, the findings of our study demonstrate that vascular closure devices are associated with a significant reduction in femoral artery access site adverse outcomes as compared to manual compression alone in patients who undergo PCI, but no significant difference among patients with diagnostic cardiac catheterization.

## Declaration of competing interest

Mr Kreutz has received prior research funding from 10.13039/501100016198Idorsia and has served as a scientific consultant for Haemonetics, Inc. Mr Ephrem has received speaking honorarium from Zoll, Inc.

## References

[1] Noori V.J., Eldrup-Jørgensen J. (2018). A ​systematic review of vascular closure devices for femoral artery puncture sites. J ​Vasc Surg.

[2] MynxGrip instructions for use. https://emea.cordis.com/content/dam/cordis/web/documents/brochure/Cordis-MynxGripVascularClosureDeviceInstructionsForUse.pdf.

[3] Angio-Seal VIP instructions for use. https://www.terumois.com/content/dam/terumo-www/global-shared/terumo-tis/en-us/product-assets/angio-seal-family/angio-seal-vip-ifu.pdf.

[4] Perclose Proglide instructions for use. https://www.accessdata.fda.gov/cdrh_docs/pdf/P960043S097D.pdf.

[5] Andrade P.B., Mattos L.A., Rinaldi F.S. (2017). Comparison of a vascular closure device versus the radial approach to reduce access site complications in non-ST-segment elevation acute coronary syndrome patients: the angio-seal versus the radial approach in acute coronary syndrome trial. Catheter Cardiovasc Interv.

[6] Martin J.L., Pratsos A., Magargee E. (2008). A ​randomized trial comparing compression, Perclose Proglide and Angio-Seal VIP for arterial closure following percutaneous coronary intervention: the CAP trial. Catheter Cardiovasc Interv.

[7] Essibayi M.A., Cloft H., Savastano L.E., Brinjikji W. (2021). Safety and efficacy of Angio-Seal device for transfemoral neuroendovascular procedures: a systematic review and meta-analysis. Interv Neuroradiol.

[8] Iannaccone M., Saint-Hilary G., Menardi D. (2018). Network meta-analysis of studies comparing closure devices for femoral access after percutaneous coronary intervention. J ​Cardiovasc Med (Hagerstown).

[9] Nikolsky E., Mehran R., Halkin A. (2004). Vascular complications associated with arteriotomy closure devices in patients undergoing percutaneous coronary procedures: a meta-analysis. J ​Am Coll Cardiol.

[10] NCDR CathPCI registry v4.4 Coder's data dictionary. https://www.ncdr.com/WebNCDR/docs/default-source/public-data-collection-documents/cathpci_v4_codersdictionary_4-4.pdf?sfvrsn=b84d368e_2.

[11] Lee M.S., Applegate B., Rao S.V., Kirtane A.J., Seto A., Stone G.W. (2014). Minimizing femoral artery access complications during percutaneous coronary intervention: a comprehensive review. Catheter Cardiovasc Interv.

[12] Sandoval Y., Burke M.N., Lobo A.S. (2017). Contemporary arterial access in the cardiac catheterization laboratory. JACC Cardiovasc Interv.

[13] Ben-Dor I., Sharma A., Rogers T. (2021). Micropuncture technique for femoral access is associated with lower vascular complications compared to standard needle. Catheter Cardiovasc Interv.

[14] Rickli H., Unterweger M., Sütsch G. (2002). Comparison of costs and safety of a suture-mediated closure device with conventional manual compression after coronary artery interventions. Catheter Cardiovasc Interv.

[15] Smilowitz N.R., Kirtane A.J., Guiry M. (2012). Practices and complications of vascular closure devices and manual compression in patients undergoing elective transfemoral coronary procedures. Am J Cardiol.

[16] Resnic F.S., Majithia A., Marinac-Dabic D. (2017). Registry-based prospective, active surveillance of medical-device safety. N ​Engl J Med.

[17] Chhatriwalla A.K., Amin A.P., Kennedy K.F. (2013). Association between bleeding events and in-hospital mortality after percutaneous coronary intervention. JAMA.

[18] Collet J.P., Thiele H., Barbato E. (2021). 2020 ESC guidelines for the management of acute coronary syndromes in patients presenting without persistent ST-segment elevation. Eur Heart J.

[19] Lawton J.S., Tamis-Holland J.E., Bangalore S. (2022). 2021 ACC/AHA/SCAI guideline for coronary artery revascularization: executive summary: a report of the American College of Cardiology/American Heart Association Joint Committee on clinical practice guidelines. J ​Am Coll Cardiol.

[20] Le May M., Wells G., So D. (2020). Safety and efficacy of femoral access vs radial access in ST-segment elevation myocardial infarction: the SAFARI-STEMI randomized clinical trial. JAMA Cardiol.

